# Epidemiology of postoperative bleeding after endoscopic nasal and sinus surgery and factors associated with bleeding

**DOI:** 10.1007/s00405-023-08377-z

**Published:** 2023-12-12

**Authors:** Tomotaka Hemmi, Kazuhiro Nomura, Yuta Kobayashi, Yuki Numano, Ryoukichi Ikeda, Mitsuru Sugawara, Yukio Katori

**Affiliations:** 1 Department of Otolaryngology, Tohoku Kosai Hospital, 2-3-11 Kokubun-cho, Aoba-Ku, Sendai, 980-0803 Japan; 2 Department of Otolaryngology, Head and Neck Surgery, Tohoku University School of Medicine, 1-1 Seiryo-machi, Aoba-Ku, Sendai, 980-8574 Japan; 3 Department of Otolaryngology-Head and Neck Surgery, Iwate Medical University School of Medicine, 1-1-1 Yahaba-cho, Shiwa-gun, Iwate, 028-3695 Japan

**Keywords:** Nasal, Sinus, Endoscopic surgery, Postoperative bleeding

## Abstract

**Purpose:**

Endoscopic nasal and sinus surgery is a surgical procedure frequently performed by otolaryngologists. Postoperative bleeding is detrimental to both healthcare providers and patients. We investigated the epidemiology of postoperative bleeding during endoscopic nasal and sinus surgery and explored possible bleeding triggers.

**Methods:**

We evaluated the patients who underwent endoscopic nasal and sinus surgery. Data regarding the age, sex, presence of hypertension, and abnormal coagulability, including oral anticoagulants, diagnoses, operative procedures, intraoperative use of drills and blood loss, and postoperative antimicrobial administration of eligible patients, were extracted from medical records and retrospectively reviewed.

**Results:**

One hundred and eighty-six patients underwent endoscopic nasal or sinus surgery during the study period, and postoperative bleeding occurred in 9 patients (4.8%). Posterior nasal neurotomy (PNN) was the procedure most likely to cause postoperative bleeding (4 surgeries, 13.3%). Postoperative antimicrobial administration significantly reduced the incidence of postoperative bleeding (*p* = 0.04).

**Conclusions:**

Postoperative bleeding requiring intervention occurs in 4.8% of cases, and PNN is associated with a high risk of postoperative bleeding. Wound infection is a potential cause of postoperative bleeding, and antimicrobial administration should be considered in addition to local treatment.

## Introduction

Endoscopic nasal and sinus surgery is a surgical procedure frequently performed by otolaryngologists. Although safer and more effective techniques and innovations have been reported daily, it is difficult to reduce the number of adverse events caused by surgery to zero, and postoperative bleeding is detrimental to both healthcare providers and patients.

There are few reports on postoperative bleeding after endoscopic nasal and sinus surgery [1, 2], and the factors contributing to postoperative bleeding are still debatable. In addition, it is crucial to understand when postoperative bleeding is most likely to occur and the types of procedures that are most likely to cause postoperative bleeding to provide more appropriate medical care.

This study investigated the epidemiology of postoperative bleeding during endoscopic nasal and sinus surgery and explored possible bleeding triggers.

## Materials and methods

### Subjects

We evaluated patients who visited and underwent endoscopic nasal or sinus surgery at the Otolaryngology Department of Tohoku Kosai Hospital in Japan between October 2022 and April 2023. Data regarding the age, sex, presence of hypertension, and abnormal coagulability, including oral anticoagulants, diagnoses, operative procedures, intraoperative use of drills and blood loss, and postoperative antimicrobial administration in eligible patients, were extracted from medical records and retrospectively reviewed.

This study was approved by the Ethics Committee of Tohoku Kosai Hospital (kkrtohoku-202307otor_S1-2_01).

### Surgical procedure and medical treatment

Each procedure was performed under general or local anesthesia by board-certified otorhinolaryngologists. Patients with chronic rhinosinusitis (CRS) underwent functional endoscopic sinus surgery (FESS). In patients with hypertrophic rhinitis, we performed inferior turbinectomy, in which the mucosal epithelium was preserved, the inferior turbinate was reduced, and the bone was further lateralized [3]. Septoplasty was performed in all patients with septal deviation, and the incisions were sutured. Posterior nasal neurotomy (PNN) was performed in patients with severe allergic rhinitis. The sphenopalatine artery (SPA) was not ligated, and only the nerve fibers were amputated. Patients with malignant tumors underwent en bloc resection with a safety margin, whereas patients with benign tumors, including inverted papilloma, underwent en bloc or piecemeal resection. For both inflammatory and neoplastic lesions, Draf surgery was performed for frontal sinus lesions [4], and endoscopic modified medial maxillectomy (EMMM) or direct approach to the anterior and lateral parts of the maxillary sinus with an endoscope (DALMA) was performed for maxillary sinus lesions, if necessary [5, 6].

Although packing materials varied among surgeons, the materials that needed to be removed were removed on the second postoperative day in all cases, and nasal rinses were started on the same day. After septoplasty, the nasal septum was compressed from both sides with 1-mm silicone sheets and fixed with nylon threads, which were removed up to 1 month after surgery. Patients undergoing surgery under general anesthesia received cefazolin sodium (CEZ) (2 g intravenously, depending on body weight, twice a day) on the day of surgery. For patients undergoing sinus surgery, clarithromycin (CAM) (200 mg orally, twice daily) was administered from the day after surgery until day 7. Tranexamic acid (1 g, intravenously) was administered intraoperatively in all patients to reduce intraoperative blood loss and the risk of perioperative bleeding.

### The evaluation of postoperative bleeding

In this study, postoperative bleeding was defined as that not achieved by observation or administration of a hemostatic agent, and requiring medical attention and treatment. The number of days from the date of surgery, the site of bleeding, the procedure that was presumed to have caused the bleeding, and treatment details were collected.

### Statistical analyses

For descriptive statistics, values are presented as the mean ± standard deviation. The interval estimation of the population proportion was calculated and presented as a 95% confidence interval (CI). Differences in characteristics between subjects with and without postoperative bleeding were evaluated using Fisher’s exact test for categorical variables and the Mann**–**Whitney *U* test for continuous variables.

All statistical analyses were performed using EZR, which was used for R. More precisely, it is a modified version of the R commander designed to add statistical functions frequently used in biostatistics [7].

## Results

### Subjects’ characteristics

One hundred and eighty-six patients underwent endoscopic nasal or sinus surgery during the study period. The demographic data of the patients are shown in Table 1. The majority of patients had CRS (64.5%), deflected nasal septum (61.2%), and hypertrophic rhinitis (60.7%). This study included sinus fungus balls and odontogenic maxillary sinusitis in CRS patients. ESS, inferior turbinectomy, and septoplasty accounted for the majority of operative procedures performed.

**Table 1:** Patients’ information, diagnoses, and operative procedures

Item	Value
Patients (*n*)	186
Age (years)	48.7 ± 16.6
Male/female	130/56
Presence of hypertension Y/N	51/135
Presence of abnormal coagulability Y/N	8/178
Diagnosis (number of patients)	
Chronic rhinosinusitis	120
Deflected nasal septum	114
Hypertrophic rhinitis	113
Allergic rhinitis	15
Tumor	10
Postoperative maxillary cyst	10
Odontogenic cyst	2
Allergic fungal rhinosinusitis	1
Invasive fungal rhinosinusitis	1
Operative procedure (number of operations)	
Functional endoscopic sinus surgery	226
Inferior turbinectomy	225
Septoplasty	114
Posterior nasal neurotomy	30
EMMM	18
Draf	12
Endoscopic tumor resection	10
DALMA	2
Intraoperative blood loss (ml)	70.5 ± 110.3
Intraoperative use of drills Y/N	31/155
Postoperative antibiotics Y/N	140/46

*EMMM* endoscopic modified medial maxillectomy, *DALMA* direct approach to the anterior and lateral part of the maxillary sinus with an endoscope

### Etiology and clinical indicators of postoperative bleeding

Postoperative bleeding occurred in nine patients (4.8%). The details for each case are presented in Table 2. In most cases, bleeding was observed at least 1 week after surgery. The operative procedures thought to cause postoperative bleeding were PNN (4 out of 30 surgeries, 13.3%) (95% CI 3.8–30.7%), EMMM (2 out of 18 surgeries, 11.1%) (95% CI 1.4–34.7%), inferior turbinectomy (2 out of 225 surgeries, 0.9%) (95% CI 0.1–3.2%), and FESS (1 out of 226 surgeries, 0.4%) (95% CI 0–2.4%), and significant differences were observed among the operative procedures (*p* < 0.01). Hemostatic procedures were performed under local or general anesthesia, and no cases required blood transfusion. When a univariate analysis was performed to identify the clinical factors that could lead to postoperative bleeding, only the presence of postoperative antimicrobial administration was statistically significant (Table 3).

**Table 2:** Details of the patients with postoperative bleeding

Case	Age (years)	Sex	Diagnosis	Operative procedure	Date of postoperative bleeding	Bleeding site	Responsible operative procedure	Treatment
1	72	Female	Hypertrophic rhinitis, allergic rhinitis	Inferior turbinectomy, PNN	8	Posterior of the right middle nasal meatus	PNN	Coagulation under general anesthesia
2	23	Female	Deflected nasal septum, hypertrophic rhinitis, allergic rhinitis	Septoplasty, inferior turbinectomy, PNN	10	Posterior of the right middle nasal meatus	PNN	Packing under local anesthesia
3	61	Male	Post operative maxillary cyst	EMMM	10	Anterior of the left inferior nasal meatus	EMMM	Coagulation under local anesthesia
4	77	Female	Post operative maxillary cyst	EMMM	7	Lateral bony surface of the right nasal cavity	EMMM	Coagulation under general anesthesia
5	17	Female	CRS, hypertrophic rhinitis	FESS, inferior turbinectomy	9	Anterior of the left inferior turbinate	Inferior turbinectomy	Coagulation under local anesthesia
6	44	Male	CRS, deflected nasal septum, hypertrophic rhinitis	FESS, Septoplasty, inferior turbinectomy	7	Outside of the left ethmoidal sinus	FESS	Coagulation under local anesthesia
7	37	Male	Deflected nasal septum, hypertrophic rhinitis	Septoplasty, inferior turbinectomy	7	Anterior of the right inferior turbinate	Inferior turbinectomy	Packing under local anesthesia
8	38	Male	Deflected nasal septum, hypertrophic rhinitis, allergic rhinitis	Septoplasty, inferior turbinectomy, PNN	8	Posterior of the left middle nasal meatus	PNN	Packing under local anesthesia
9	33	Male	Deflected nasal septum, hypertrophic rhinitis, allergic rhinitis	Septoplasty, inferior turbinectomy, PNN	5	Posterior of the right middle nasal meatus	PNN	Coagulation under local anesthesia

*CRS* chronic rhinosinusitis, *EMMM* endoscopic modified medial maxillectomy, *FESS* functional endoscopic sinus surgery, *PNN* posterior nasal neurotomy

**Table 3:** Associations of clinical factors with postoperative bleeding

Factors	Postoperative bleeding		*p* value
	No	Yes	
Hypertension			
No	129	6	0.7^a^
Yes	48	3	
Abnormal coagulability			
No	171	7	0.05^a^
Yes	6	2	
Intraoperative use of drills			
No	148	7	0.64^a^
Yes	29	2	
Postoperative antimicrobial administration			
No	41	5	0.04^a^
Yes	136	4	
Intraoperative blood loss (ml)	71.6 ± 112.1	49.4 ± 66.7	0.53^b^

^a^Fisher’s exact test

^b^Mann–Whitney *U* test

## Discussion

The frequency and typical timing of postoperative bleeding during various surgical procedures were determined. Although it is a rare and essentially nonfatal adverse event, efforts should be made to reduce postoperative bleeding to zero whenever possible. A previous study reported a postoperative bleeding frequency of 8.4%, which is generally similar to the results of the present study, but with no marked difference in bleeding frequency according to surgical technique [1]. This is because instruments and workmanship differ among facilities, even for the same surgical procedure with the same name.

PNN suppresses secretagogue motor and neurogenic inflammation by severing the parasympathetic and sensory nerve fibers [8]. Conventional practice is to sever the SPA and nerve fibers [9], but methods to preserve the SPA to control intraoperative bleeding have been reported [10]. Although some reports indicate that PNN is a complication-free technique [11, 12], postoperative bleeding occurs at a higher rate than in other endoscopic procedures, as shown in this study. Postoperative bleeding from the surgical site should be considered, especially when the SPA is spared, as blood flow to its branches is preserved. PNN is a common procedure in Japan and a good option for patients who do not show improvement with conservative treatment. However, it is important to keep in mind that there is a risk of postoperative bleeding, especially for patients who cannot tolerate this risk. In cases of EMMM, Nakayama et al. reported postoperative bleeding as a complication [5]. EMMM is a very effective technique for managing maxillary sinus lesions; however, postoperative bleeding can occur. In inferior turbinectomy, preservation of the mucosal epithelium prevents postoperative bleeding and crust adhesion [3]. Our department also performed surgery according to this method; however, sutures at the mucosal incision were omitted in cases that resulted in postoperative bleeding. Surgeons must acquire the skill to suture the nasal cavity properly and not skimp on the time and effort required to place sutures. Postoperative bleeding is reported to occur in 6% of patients who undergo FESS, and anticoagulant medications, hypertension, and high Lund–Mackay scores are significant risk factors [2]. Although these previous results differ somewhat from those of the present study, those studies did not examine the effect of postoperative antimicrobial use on postoperative hemorrhaging. Increased blood flow associated with postoperative infection may be a factor contributing to postoperative bleeding.

In animal experiments using rabbits, it was observed that the damaged nasal mucosa showed inflammatory cell infiltration after roughly 1 week, and epithelialization was completed in 1 month [13]. Most postoperative bleeding occurred within 1 week after surgery, suggesting that problems in the wound healing process were a factor in bleeding. Wound infections may cause bleeding, as bleeding was observed at a significant frequency in patients who did not receive postoperative oral antimicrobial administration. In addition to local treatment, such as cleaning and rinsing after surgery, antimicrobial administration may reduce postoperative bleeding. Prospective studies on septoplasty have reported that antimicrobial administration is unnecessary [14]. However, as the present study revealed that septoplasty is not prone to postoperative bleeding, it is necessary to examine various procedures to determine whether or not antimicrobials should be administered after endoscopic nasal and sinus surgery.

Several limitations associated with the present study warrant mention. First, the patient population in this study included those who visited a hospital in a specific region of Japan. Therefore, the results of this study may differ across different areas and periods. Second, only univariate statistical analysis was performed because of the sample size. However, postoperative bleeding is a rare complication, this problem cannot be easily resolved. This study is valuable for informing surgeons about the types of procedures prone to postoperative bleeding and when they are most likely to occur.

## Conclusion

Postoperative bleeding requiring intervention occurs in 4.8% of cases, and PNN is associated with a high risk of postoperative bleeding. Wound infection is a potential cause of postoperative bleeding, and antimicrobial administration should be considered in addition to local treatment.
